# Use of neoadjuvant chemotherapy prior to radical hysterectomy in cervical cancer: monitoring tumour shrinkage and molecular profile on magnetic resonance and assessment of 3-year outcome

**DOI:** 10.1038/sj.bjc.6601870

**Published:** 2004-05-25

**Authors:** N M deSouza, W P Soutter, G Rustin, M M Mahon, B Jones, R Dina, G A McIndoe

**Affiliations:** 1 Department of Imaging, Hammersmith Hospital, DuCane Road, London W12 0HS, UK; 2 Departments of Obstetrics and Gynaecology, Hammersmith Hospital, DuCane Road, London W12 0HS, UK; 3 Department of Histopathology, Hammersmith Hospital, DuCane Road, London W12 0HS, UK; 4 Department of Radiation Oncology, Hammersmith Hospital, DuCane Road, London W12 0HS, UK; 5 The Robert Steiner MR Unit, Hammersmith Hospital, DuCane Road, London W12 0HS, UK; 6 Department of Medical Oncology, Mount Vernon Hospital, Middlesex, UK

**Keywords:** cervical cancer, neoadjuvant chemotherapy, magnetic resonance imaging, magnetic resonance spectroscopy, outcome

## Abstract

The objective of this study is to assess tumour response to neoadjuvant chemotherapy prior to radical hysterectomy in cervical cancer using magnetic resonance (MR) to monitor tumour volume and changes in molecular profile and to compare the survival to that of a control group. Eligibility included Stage Ib–IIb previously untreated cervical tumours >10 cm^3^. Neoadjuvant chemotherapy in 22 patients (methotrexate 300 mg m^−2^ (with folinic acid rescue), bleomycin 30 mg m^−2^, cisplatin 60 mg m^−2^) was repeated twice weekly for three courses and followed by radical hysterectomy. Post-operative radiotherapy was given in 14 cases. A total of 23 patients treated either with radical surgery or chemoradiotherapy over the same time period comprised the nonrandomised control group. MR scans before and after neoadjuvant chemotherapy and in the control group documented tumour volume on imaging and metabolites on *in vivo* spectroscopy. Changes were compared using a paired *t*-test. Survival was calculated using the Kaplan–Meier method. There were no significant differences between the neoadjuvant chemotherapy and control groups in age (mean, s.d. 43.3±10, 44.7±8.5 years, respectively, *P*=0.63) or tumour volume (medians, quartiles 35.8, 17.8, 57.7 cm^3^ *vs* 23.0, 15.0, 37.0 cm^3^, respectively, *P*=0.068). The reduction in tumour volume post-chemotherapy (median, quartiles 7.5, 3.0, 19.0 cm^3^) was significant (*P*=0.002). The reduction in –CH_2_ triglyceride approached significance (*P*=0.05), but other metabolites were unchanged. The 3-year survival in the chemotherapy group (49.1%) was not significantly different from the control group (46%, *P*=0.94). There is a significant reduction in tumour volume and –CH_2_ triglyceride levels after neoadjuvant chemotherapy, but there is no survival advantage.

Neoadjuvant chemotherapy has been advocated prior to radical hysterectomy in locally advanced cervical carcinoma because it may serve to control micrometastatic disease and so improve survival (Sardi *et al*, 1997; Benedetti-Panici *et al*, 1998; Hwang *et al*, 2001). In addition, it offers the potential to reduce tumour volume and thus improve surgical resectability (Eddy *et al*, 1995). However, the implementation of a chemotherapeutic regime delays surgery by 6 weeks, with the potential for transforming a surgically resectable tumour to one that is not. The purpose of this study was to assess tumour response to neoadjuvant chemotherapy prior to radical hysterectomy in cervical cancer using magnetic resonance imaging to monitor tumour volume and magnetic resonance spectroscopy to assess change in molecular profile. The survival was compared to that of a control group of equivalent stage and tumour volume who were treated conventionally either with radical surgery or chemoradiotherapy over the same time period.

## PATIENTS AND METHODS

All women presenting to our gynaecological oncology surgical centre and assessed as being potential candidates for radical hysterectomy underwent magnetic resonance imaging using a conventional plus an endovaginal technique. Over the 57-month period March 1996–December 2000, all patients with tumour volume >10 cm^3^ and Stage Ib–IIb were included in this study: those from one oncological referral centre were offered neoadjuvant chemotherapy prior to radical hysterectomy plus adjuvant radiotherapy as indicated, while those from all other referral units were offered radical hysterectomy alone (*n*=5), radical hysterectomy followed by adjuvant radiotherapy (*n*=3), chemoradiotherapy (*n*=13; Green *et al*, 2001) or lymph node dissection and radiotherapy (*n*=2) as indicated. This reflected the clinical practice at these centres at that time. Neoadjuvant chemotherapy consisted of methotrexate 300 mg m^−2^ (with folinic acid 15 mg 12 h for six doses as rescue after 24 h), bleomycin 30 mg m^−2^ and cisplatin 60 mg m^−2^ repeated twice weekly for three courses. Radical hysterectomy was performed within 7 days of completion of neoadjuvant chemotherapy. Adjuvant radiotherapy consisted of 45 Gy in 25 fractions to a volume that encompassed the top of the sacroiliac joints to the bottom of the obturator foramen in all cases. Chemoradiotherapy regime in the control group consisted of 50 Gy fractionated over 5−5.5 weeks with cisplatin 40 mg m^−2^ weekly over that time period, followed by high dose rate intracavitary brachytherapy tailored to the individual (usually 14 Gy in two insertions). Three patients in the control group received additional radiation to the para-aortic nodes.

### Magnetic resonance imaging

The endovaginal receiver coil was of solenoid geometry design, 37 mm in diameter, mounted on a Delrin™ ring. Details of the endovaginal coil have been described previously (deSouza *et al*, 1996). The coil was inserted into the vagina and positioned around the cervix by manipulation of the handle, then immobilised by an external clamp. All patients were able to tolerate the coil for the length of the examination (30–40 min) and in no case was the examination terminated because of patient discomfort.

Imaging was performed either at 0.5 or 1.5 T (Apollo or Eclipse, Marconi Medical Systems, Highland Heights, OH, USA) using transverse to the cervix T1-weighted spin echo (SE 720-820/20 (TR/TE) ms), and sagittal T2-weighted fast spin-echo (FSE 2500/80 (TR/TE) ms) sequences. Contiguous slices 2–3 mm thick were acquired with a 192 × 256 matrix, two to four signal averages and a 12-cm field of view. On completion of the endovaginal study, the coil was removed. An external four-channel pelvic phased array coil (Picker International, Highland Heights, OH, USA) was then used to acquire sagittal and transverse to the cervix T2-weighted FSE images with 6 mm contiguous slices, a 192 × 256 matrix, two to four signal averages and a 25–35 cm field of view at 0.5 T. Additional coronal STIR sequences were also done for assessment of pelvic lymphadenopathy.

### *In vivo*^1^H-MRS

T_2_-weighted images sagittal and transverse to the cervix were used to position a 3.4 cm^3^ voxel centrally within the cervical mass. Shimming on the voxel was done using a point-resolved spectroscopy sequence (PRESS) technique to give the minimum water line width at half height. This value was recorded. Localisation and acquisition of ^1^H-MR spectra were performed using the PRESS technique (TR, 1600 ms, TEs, 68, 135 and 270 ms). The total acquisition time for each echo time was 3.8 min. Water suppression was obtained with three initial chemical shift selective saturation (CHESS) pulses. Resonance peaks were all phased to choline-containing compounds and their chemical shifts assigned relative to that of the total choline peak at 3.2 p.p.m.

### Data analysis

#### MR imaging studies

The presence of a mass within the cervix was recorded and tumour volumes measured on the T_2_-weighted sagittal images by drawing a region of interest around the intermediate signal-intensity mass of tumour on each slice and multiplying the total area by slice thickness. This was done three times in each patient by a single observer (NdS) and a <1% variation in volume obtained between the readings. Previous correlation of such measurements with volumes measured on pathology specimens has shown excellent agreement (Wagenaar *et al*, 2000).

#### *In vivo* MRS studies

The voxel of spectroscopic data acquisition was displayed on consecutive T_2_-weighted sagittal slices. The presence of triglyceride-CH_2_ (1.3 p.p.m.) and -CH_3_ (0.9 p.p.m.) resonances, choline-containing compounds (Cho) (3.2 p.p.m.), creatine plus phosphocreatine (Cr) (3.0 p.p.m.) and 2 p.p.m. peaks was noted. In some cases, the triglyceride signals were observed to be markedly out of phase compared with the choline resonance. As there was no evidence that this was due to a first-order phase shift, it was assumed that this represented contamination from triglyceride signals outside the voxel due to imperfect slice select pulses, compounded by patient motion. When the water line width at half height was <15 Hz and spectral quality was adequate, peak area of choline at TE 135 ms was computed using the scanner software. Owing to the difficulty in determining absolute concentrations, tissue water was used as an internal standard. As the concentration of water in normal and cancer tissue is uniform to a first-order approximation (Mahon *et al*, 2004), contamination from out-of-voxel water was not considered. Peak areas of choline, CH_2_ and 2 p.p.m. peak were analysed relative to the estimated peak area of the T_2_ corrected unsuppressed water signal from the voxel and expressed as arbitrary units (a.u.).

### Statistics

Statistical analysis was performed using SPSS, version 10.1 for Windows. For tumour volumes, as the data were not normally distributed, volumes in the neoadjuvant chemotherapy *vs* those in the conventionally treated control group were compared with the Mann–Whitney *U* test. The reduction in tumour volume before and after neoadjuvant chemotherapy was compared using a paired *t*-test. For *in vivo* MRS data, the levels of resonances in phase with choline of triglyceride-CH_2_ Cho, and signal at 2 p.p.m. before and after a 6-week course of neoadjuvant chemotherapy were compared using a paired *t*-test. Survival was calculated by the product limit method of Kaplan and Meier (Statistica version 5.0, Statsoft, Tulsa, USA).

## RESULTS

In all, 22 patients aged 28–60 years (mean, s.d. 43.3±10 y) were included in the neoadjuvant chemotherapy arm (18 stage Ib >10 cm^3^, 4 stage IIb) and 23 patients aged 33–69 years (mean, s.d. 44.7±8.5 y) were included in the control arm (16 stage Ib >10 cm^3^, 1 stage IIa, 6 stage IIb). There was no significant difference in age between the women treated with neoadjuvant chemotherapy prior to Wertheim's hysterectomy and those in the control group (treated with radical surgery or chemoradiotherapy) (*P*=0.63).

### MR imaging

In the neoadjuvant chemotherapy group, tumour volumes ranged from 11.5 to 70 cm^3^ pre-chemotherapy (median and quartiles 35.8, 17.8, 57.7 cm^3^, Table 1

**Table 1:** MR findings in the neoadjuvant chemotherapy *vs* control groups

**MRI features**	**Neoadjuvant chemotherapy group**	**Control group**
MRI tumour volume pre (median, quartiles)	35.8 (17.8, 57.7) cm^3^	23.0 (15.0, 37.0) cm^3^
MRI tumour volume post (median, quartiles)	7.5 (3.0, 19.0) cm^3^	
Parametrial extension %	59.1 (13/22)	56.5 (13/23)
Enlarged nodes %	77.3 (17/22)	60.9 (14/23)

). Post chemotherapy scans were done in 19 cases (three failed to attend) and tumour volumes ranged from 0.9 to 87.0 cm^3^ (median, quartiles 7.5, 3.0, 19.0 cm^3^). There was a highly significant reduction in tumour volume (median reduction 68.6 cm^3^) after treatment (*P*=0.002, Figure 1

**Figure 1:**
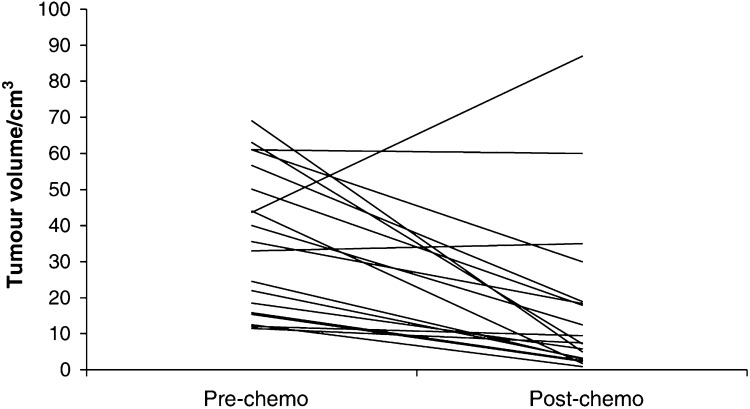
Tumour volumes pre and post neoadjuvant chemotherapy, showing response in individual cases.

). A >50% reduction in tumour volume (partial response) was seen in 14 out of 19 cases (73.7%). Tumour volume decreased after chemotherapy in 16 (84%) out of 19 cases (Figure 2

**Figure 2:**
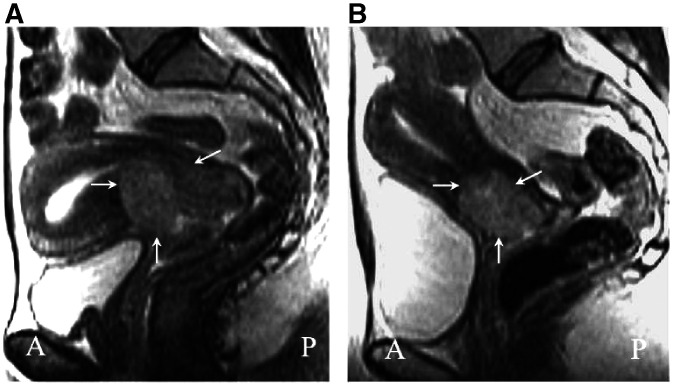
Sagittal T2-weighted fast spin-echo (FSE 3000/88 ms (TR/effective TE)) images before (**A**) and after (**B**) three cycles of neoadjuvant chemotherapy. The intermediate signal intensity tumour confined to the cervix (arrows) reduced from 50 to 18 cm^3^ in volume.

); in two cases it remained the same and in one case there was a doubling of tumour volume after 6 weeks of chemotherapy. Tumour volume in the control group ranged from 10.1 to 72 cm^3^ (median and quartiles 23.0, 15.0, 37.0 cm^3^). Tumour volumes were not significantly smaller than those in the neoadjuvant chemotherapy arm (Mann–Whitney *U*-test, *P*=0.068). Although the neoadjuvant chemotherapy group had more patients with tumour volumes greater than 40 cm^3^, our data show that volume has less impact on mortality when it exceeds 20 cm^3^ (Soutter *et al*, in press). Distribution of tumour volume in the groups is shown in Figure 3

**Figure 3:**
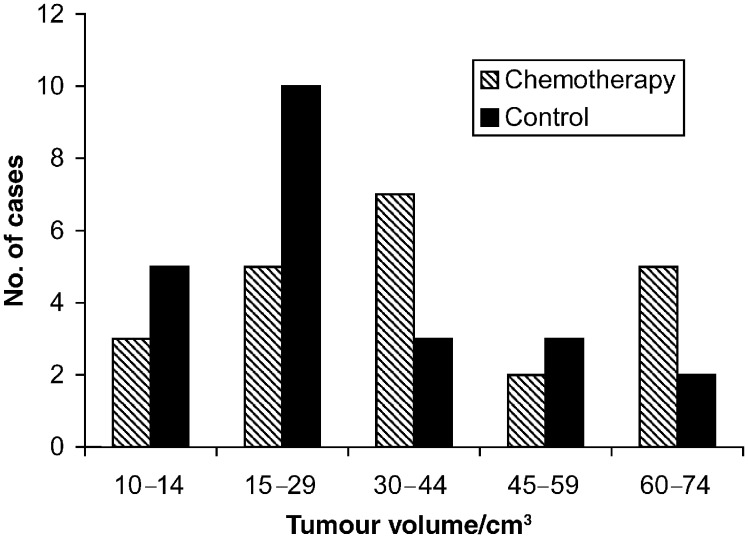
Tumour volume distribution prior to treatment in the neoadjuvant chemotherapy group and the control group.

. The characteristics of the two groups in terms of MR features (parametrial extension, enlarged lymph nodes) are shown in Table 1. The histological findings after radical hysterectomy in the two groups (parametrial extension, lymph node positivity) for the two groups are shown in Table 2

**Table 2:** Histological features in the neoadjuvant chemotherapy *vs* control groups

**Histological features**	**Neoadjuvant chemotherapy group (%)**	**Control group (%)**
*Type*		
Squamous	72.7 (16/22)	73.9 (17/23)
Adenocarcinoma	13.6 (3/22)	13.0 (3/23)
Adeno-squamous	9.1 (2/22)	4.3 (1/23)
Other	4.5 (1/22)	4.3 (1/23)
		
*Grade*		
1	4.5 (1/22)	4.3 (1/23)
2	45.5 (10/22)	34.8 (8/23)
3	50.0 (11/22)	60.9 (14/23)
		
Lymphovascular space invasion	47.6 (10/21)1 patient had RT to primary site	42.9 (6/14; 9 biopsies inadequate to assess)
Parametrial invasion	33.3 (7/21)	12.5 (1/8; 8 had RHND)
Lymph node involvement	50.0 (11/22)	40.0 (4/10; 10 had LND)

.

### MR spectroscopy

**Figure 4:**
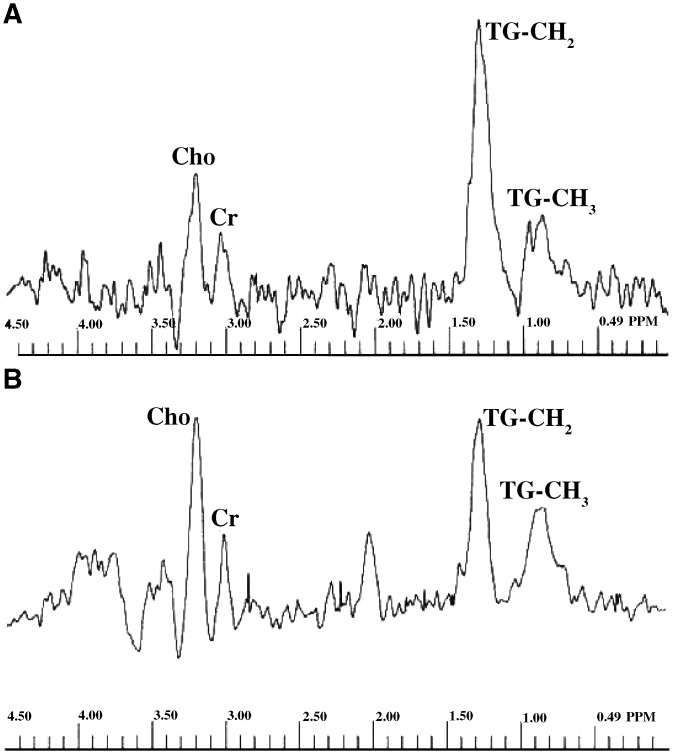
Proton magnetic resonance spectra (^1^H PRESS, TR=1600 ms; TE=135 ms) acquired from a 3.75 cm^3^ voxel within the tumour before (**A**) and after (**B**) three cycles of neoadjuvant chemotherapy. Triglyceride signal (1.3 p.p.m.) in-phase with choline-containing compounds (3.2 p.p.m.) is reduced in (**B**).

Nine of 22 patients had *in vivo* MR spectroscopy before and after neoadjuvant chemotherapy. A typical *in vivo* spectrum from these patients is illustrated and showed peaks from choline (3.2 p.p.m.), a peak at 2 p.p.m. and CH_2_ at 1.3 p.p.m. (Figure 4

). The concentration of water in the tumour pre (mean, s.d. 1.9±0.8 × 10^6^ a.u.) and post (mean, s.d. 2.6±1.7 × 10^6^ a.u.) chemotherapy was similar to a first-order approximation (*P*=0.14). Levels of choline pre and post neoadjuvant chemotherapy were 4.69±8.13 × 10^−4^ and 2.19±1.42 × 10^−4^ a.u. (mean, s.d.), respectively, but differences were not statistically significant (*P*=0.39). Levels of the 2 p.p.m. peak pre and post neoadjuvant chemotherapy were 4.78±13.15 × 10^−4^ and 1.24±1.27 × 10^−4^ a.u. (mean, s.d.), respectively, but differences were not statistically significant (*P*=0.46). Levels of the CH_2_ peak pre and post neoadjuvant chemotherapy were 3.85±5.53 × 10^−4^ and 1.23±2.4 × 10^−4^ a.u. (mean, s.d.), respectively, which just approached significance (*P*=0.05).

### Outcome

**Figure 5:**
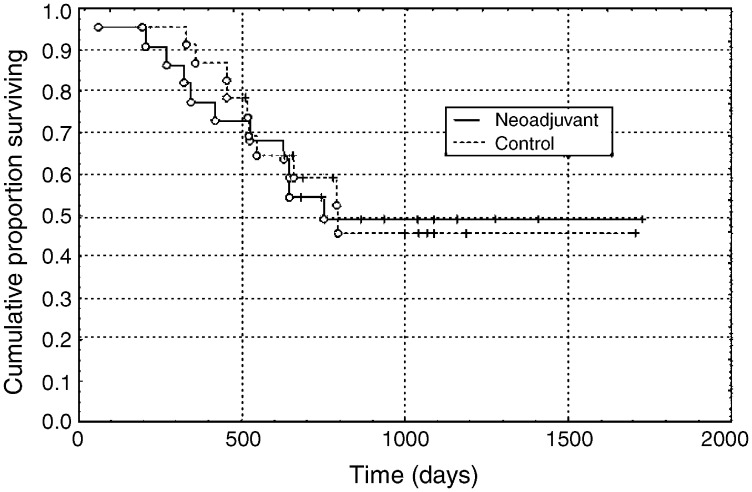
Cumulative proportions surviving in each group. The solid line shows the Kaplan–Meier survival curve for the neoadjuvant chemotherapy group and the dashed line is the survival curve for the control group. The vertical dashes are censored observations and the open symbols are deaths.

The median length of follow-up of 23 surviving patients was 1043 days (range 510–2258 days) and 22 women relapsed and died. There was no difference in 3-year survival between the women receiving neoadjuvant chemotherapy and those in the control group treated primarily with radical surgery or chemoradiotherapy (49.1 *vs* 46.0%, *P*=0.94, Figure 5

). In the neoadjuvant chemotherapy group, eight of 11 (72.7%) patients with positive lymph nodes died of disease, while only three of 11 (27.3%) patients with negative lymph nodes died of disease.

## DISCUSSION

Our study shows a reduction in tumour volume in 84% of patients following neoadjuvant chemotherapy, two patients showed no response and one patient's disease progressed. The reduction in tumour volume, following neoadjuvant chemotherapy, offers the potential of facilitating primary surgery. In addition, it may serve to control micrometastatic disease and so improve survival.

Previous studies have relied on clinical evaluation by pelvic examination with or without ultrasonography to assess the response (Eddy *et al*, 1995; Lai *et al*, 1997; Sardi *et al*, 1997; Benedetti-Panici *et al*, 1998; Hwang *et al*, 2001). Reductions in tumour volume of up to 100% with as many as 15–20% having complete clinical response after three cycles of chemotherapy (Lai *et al*, 1997; Benedetti-Panici *et al*, 1998) have been reported, but on comparison with subsequent histopathology these assessments have been shown to be inaccurate (Eddy *et al*, 1995). MR imaging using an endovaginal receiver coil allows an improved signal-to noise ratio immediately adjacent to the region of interest and provides accurate assessment of tumour volume and parametrial extension (deSouza *et al*, 2000). The median tumour volume was reduced by 79% in our study. However, our tumour volume measurements were made by drawing a region of interest around an intermediate to high signal intensity mass within the cervix, so it was not possible to distinguish treated from viable tumour. It may well be that our post-treatment measurements included non-viable tumour.

Tumour necrosis and regression are often associated with dense fibrosis, which may complicate surgical management. In our cohort, operability after neoadjuvant chemotherapy was not compromised. All patients receiving neoadjuvant chemotherapy were judged on MR imaging to be operable at the outset. No patient was subsequently found to be inoperable, either because of disease progression or because of tissue fibrosis/inflammation following chemotherapy. Paladini *et al* (1995) have reported an intra-operative complication rate of 13.3% for these tumours, with an overall post-operative complication rate of 4.4%, which was not significantly different from the reported complication rate for radical surgery without neoadjuvant chemotherapy.

The molecular profile of tumours *in vivo* may be probed using ^1^H-MR spectroscopy to non-invasively identify metabolites and other cell components that have sufficient mobility to be MR detectable (Le Moyec *et al*, 1992; Kuesel *et al*, 1994a). Previous reports have shown that the MR spectra of biopsy samples from invasive cancer of the cervix are characterised by resonances of mobile triglycerides (Delikatny *et al*, 1993). Use of an endovaginal receiver coil makes it possible to obtain *in vivo* spectra from the tumour, which would not otherwise be available. We have detected the presence of positive triglyceride-CH_2_ resonances in 74% of cervical tumours *in vivo* using this technique (Mahon *et al*, 2004). Positive triglyceride signals were not detected in normal control women. This finding was corroborated on *ex vivo* MR spectroscopy studies of uterine cervical tissue from the same women.

The origin of these triglycerides is debated. High lipid levels in glioblastoma biopsy samples have been correlated with necrosis (Kuesel *et al*, 1994b), but are also seen in tumours with no apparent necrosis (Negendank and Sauter, 1996). Levels of MR visible lipids also have been shown to be directly proportional to the number of apoptotic cells (Blankenberg *et al*, 1997). In both uni- and multivariate analysis, the apoptotic index has been shown to be a strong predictor of recurrence-free survival (Costa *et al*, 2001). It therefore may be possible to monitor non-invasively *in vivo* using MR spectroscopy parameters that predict favourable prognosis.

We did not see any significant differences in choline-containing compounds before and after chemotherapy. Choline-containing compounds differentially accumulate in normal and malignant tissue, and have been localised, as shown in *in vivo* 1 H MR spectroscopy of brain tumours (Vigneron *et al*, 2001) prostate cancer (Kurhanewicz *et al*, 1996) and breast cancer (Kvistad *et al*, 1999). Although these studies have shown that the malignant regions have elevated total choline resonances at 3.2 p.p.m. compared to normal tissue, our *in vivo* results show no significant differences between the levels of total choline detected in cancer compared with normal (Mahon *et al*, 2004), and no statistically significant reduction in choline-containing compounds were seen after neoadjuvant chemotherapy. Partial volume effects related to tissue heterogeneity within the voxel are likely to be responsible.

Survival in both the neoadjuvant chemotherapy group and the control group was equally poor. Lymph node involvement in both groups was twice that was quoted for similar stage tumours in the literature. In other reports, for tumours clinically assessed as >4 cm diameter, a 11–25% lymph node involvement resulted in overall 5-year survival figures between 82 and 74% (Eddy *et al*, 1995; Sardi *et al*, 1997; Benedetti-Panici *et al*, 1998; Hwang *et al*, 2001). Similarly, a randomised study by Chang *et al*. (2000), comparing neoadjuvant chemotherapy followed by radical hysterectomy with radical radiotherapy in bulky stage I disease, showed a 2-year cumulative survival of 81% for the neoadjuvant chemotherapy arm, but no significant difference between that and the control arm in disease-free or overall survival. The lower survival figures in both our groups reflect the selection of a poorer prognostic group at the outset in terms of tumour burden and lymph node metastases. A more recent phase II study of 441 stages Ib2-III cervical cancers randomised to neoadjuvant chemotherapy plus radical surgery *vs* radical radiotherapy showed an overall 5-year survival of 64.7% for the Stage Ib2–IIb subgroup in the neoadjuvant chemotherapy arm (Benedetti-Panici *et al*, 2002). This is more in keeping with our data.

Neoadjuvant chemotherapy has also been advocated for patients younger than 50 years (mean 38 years), where an overall 5-year survival of 84% was seen in bulky Ib and IIa tumours (Aoki *et al*, 2001). The incidence of parametrial invasion and lymph node invasion in these patients was 29%, and 29% invaded less than two-thirds into the cervical stromal on MR imaging. In our cohort, two of seven patients over 50 years of age died of disease by 2 years, while eight of 15 patients under the age of 50 died of disease by 2 years, indicating that, in our patients with large tumour volumes, parametrial extension and lymphadenopathy on imaging, age was probably not a significant factor in determining survival.

The high response rates to cisplatin-based regimes in phase II studies (Eifel, 2000) have led to randomised trials comparing neoadjuvant chemotherapy plus primary treatment with primary treatment alone. Regimes described vary from single agent cisplatin 40 mg m^−2^ to double or triple agent therapy combining cisplatin 43–80 mg m^−2^ with bleomycin 15–30 mg m^−2^ and with the addition of vinblastine 4 mg m^−2^ or vincristine 1 mg m^−2^. Numbers of cycles varied between two and six, with reports of continued response up to five cycles (Hwang *et al*, 2001). We used a regimen from Benedetti-Panici *et al* (1991), modified from three times weekly to twice weekly, with a reduced dose of cisplatin. This regimen causes nausea, vomiting and fatigue, but no major haematological toxicity, provided the folinic acid is taken on time.

Our study shows that, although there is a significant reduction in tumour volume and –CH_2_ triglyceride levels after neoadjuvant chemotherapy, there is no survival advantage. However, in our small non-randomised cohort, the prognostic factors were not entirely balanced: although not significant, the neoadjuvant group had larger tumours, a higher prevalence of lymphovascular invasion, parametrial invasion and enlarged nodes, which, taken together, suggest that they may have been a slightly worse prognostic group. An EORTC trial (55994) is comparing neoadjuvant chemotherapy followed by radical surgery with chemoradiation, in order to determine survival benefit.
